# Ramadan fasting intentions among pregnant women in Lebanon

**DOI:** 10.1186/s42506-023-00148-2

**Published:** 2024-01-17

**Authors:** Chaza Alaeddine, Jim Schreiber, Mohamed E. K. Amin

**Affiliations:** 1 Faculty of Pharmacy, Beirut Arab University, Beirut, Lebanon; 2 School of Nursing, Duquesne University, Pittsburgh, USA; 3 Faculty of Pharmacy, Alamein International University, El Alamein, Egypt

**Keywords:** Religious fasting, Ramadan, Pregnancy, Theory of planned behavior

## Abstract

**Background:**

According to Islam’s teachings, women are religiously exempt from fasting during pregnancy if a woman is concerned about her health or that of the fetus. This study assesses the intentions of pregnant women to fast during Ramadan and evaluates the contribution of items derived from the theory of planned behavior (TPB) in predicting these intentions.

**Methods:**

A cross-sectional survey was carried out in Arabic on a convenience sample of 181 pregnant women in Lebanon using a mixture of in-person (46), telephone (31), and online recruitment (104) techniques from February to April 2020. An Exploratory Bayes Tree Analysis was done to examine which TPB items appeared to separate the intention to fast in the best possible way. Then, an ordinal regression was completed followed by a latent class analysis to examine specific classes of participants that could be determined based on the regression results.

**Results:**

Overall, 58% of participants had the intention to fast all days of Ramadan, 22% had the intention to fast some days and 20% did not intend to fast for any duration. A model was run with perceptions of physical ability, Islam guidance, husband’s opinion importance, mother’s opinion beliefs, and impact on general health as predictors (*R*^2^ = 0.74). A four-cluster model was chosen as the most parsimonious one in interpretation, where classes one and two included the groups of women who intended to fast month-long with differences in predictors. Class three represented the group of women who did not have the intention to fast and the final class represented the group of women who had the intention to fast some days of the month. The women’s belief in their physical ability to fast and the opinion of the pregnant women’s mothers were very important in deciding the participants’ intention to fast.

**Conclusions:**

Items derived from TPB constructs helped in producing a model predicting women’s intention to fast during Ramadan. Educational messages and interventions related to fasting while pregnant may be delivered by individuals with legitimacy among pregnant women such as those viewed by the target population as powerful motherly figures in their communities.

## Introduction

Ramadan, the ninth month in the lunar calendar, represents a special religious occasion for 1.6 billion Muslims around the world [1]. Fasting during the holy month is one of the five pillars of Islam, which entails total abstinence from eating, drinking, smoking, and even taking oral medications from dawn to sunset [2]. Per Islam’s teachings that urge worshipers to maintain good health and avoid harm, several exemptions from fasting are applied. Concerning the studied topic, according to most interpretations of the Quran, women are exempted from fasting if they are concerned about their own or their baby’s health. Moreover, many interpretations of verse 184 of Surat al Baqara require pregnant women who decide not to fast to make up for the non-fasted days at a later point, when they are no longer pregnant or breastfeeding [2]. To that end, the literature indicates that a significant proportion of Muslim pregnant women choose to fast [3, 4] with studies from different countries indicating that the proportion of fasting among Muslim pregnant women varied from 30 to 88%. Furthermore, the percentage of women fasting for a minimum of 15 days falls within the range of 40 to 65% [5–9].

The alteration in meal timing, meal frequency, food quantity and composition, and difference in sleeping patterns associated with fasting may lead to stressful events and put the pregnant woman and her fetus at risk [10, 11]. The most common side effects that appeared in the pregnant fasted women were hyperemesis and dehydration status which in turn could lead to an elevated risk of developing urinary tract infections [10, 12]. Moreover, several studies have discussed the medium to long-term effects of fasting on glucose-homeostasis, lipids panel, and the occurrence of premature deliveries [13, 14]. Concerning the effect of fasting on the fetus’s health, a review of the literature found that intrauterine development mainly represented by the amniotic fluid index, length of the femur, and the estimated weight of the fetus is not affected by fasting [15]. Furthermore, the effect of fasting during pregnancy on the biophysical and biochemical parameters of the fetus, such as fetal heart rate, amniotic fluid level, bladder volume, and breathing pattern, is still not well understood. Nevertheless, several studies found that fasting did not have significant adverse effects on these parameters [16, 17]. A systematic review found that few studies of average quality found a significant negative effect of Ramadan fasting for pregnant mothers on fetal growth or birth indices and concluded that the association between Ramadan fasting and the health outcomes of offspring is not supported by strong evidence [18]. In a more recent review, available studies pointed to negative associations between maternal Ramadan fasting and neonatal weight, amniotic fluid index, preterm birth, and growth parameters. It is worth noting, however, that the inadequate quality of available studies remains a significant consideration [19]. Currently, there is insufficient and controversial data about the cognitive and long-term effects on the health of the baby of the mother who observed Ramadan fasting [18, 19].

Communication between healthcare professionals and pregnant women is an important factor in providing the best maternal service [20]. Healthcare professionals should play an important role in enhancing women’s knowledge and shaping their perceptions of fasting during pregnancy [6]. Obstetricians, in particular, should acknowledge both the perceptions of Muslim women as well as the effect of fasting on the health of the mother and her fetus. However, studies indicate significant communication gaps between women who consider fasting during Ramadan and health professionals [4, 5, 21].

Following the literature review, we identified several gaps that this study addresses. Those gaps include theoretical orientation and framing of the dependent variable (intention of the future as compared to past fasting behavior). We could not identify any study assessing perceptions of pregnant women towards fasting based on a health behavior theory. Such an approach would help in guiding the selection of included variables, interpreting the data, and proposing explanations of women’s intention to fast. It would also help guide stakeholders to plan future interventions tailored to Muslim pregnant women. Most of the earlier studies asked women to report their fasting behavior in retrospect after Ramadan has elapsed [3–6]. Assessing the intention to fast provides the opportunity to avoid or reduce the effect of recall or selection bias that would be more problematic when collected data is in retrospect. Finally, the setting of this study is distinctive since Lebanon’s diversity in population and religious composition would provide an interesting model. This model would add valuable insights to the existing literature which has mostly focused on settings with either a predominantly Muslim majority or minority within the studied population.

This study aimed to assess the intentions of pregnant women to fast during Ramadan and evaluate the contribution of items derived from the theory of planned behavior (TPB) constructs in predicting the intentions of pregnant women to fast during Ramadan.

## Methods

### Theoretical framework

The TPB is used to predict the intentions of individuals to engage in a certain behavior in a specific context [22]. It is composed of four main constructs: attitude, subjective norm, perceived behavioral control, and the associated behavioral intention. According to TPB, attitudes (feelings about a behavior), subjective norms (perception of whether important people perform and approve of the behavior or not), and perceived behavioral control (perception of the difficulty of performing a behavior) determine the individual’s behavioral intention (plan to perform the behavior) and consequently determine the likelihood of the individual carrying out that specific behavior.

### Instrument construction

A quantitative cross-sectional survey design was employed. The instrument was designed in the Lebanese Arabic dialect to improve comprehension and reduce the cognitive burden for participants. It consisted of items addressing the dependent variable (women’s intention to fast) followed by the independent variables and ending with demographic and other characteristics of participants. Dependent and independent variables were designed as follows:

### Dependent variable

A *woman’s intention to fast* was assessed using one item. Participants were asked whether they planned to fast during Ramadan. Potential answers were “I am not planning to fast”, “I am planning to fast some days” or “I am planning to fast all days”.

### Independent variables

The *attitude towards the fasting effect* predictor variable was operationalized as the woman’s perception of the impact (benefit or harm) on themselves and the fetuses if they fast. Similarly, the *perceived ability to fast* predictor variable was operationalized as a woman’s perceived ability to fast during Ramadan while pregnant. In addition to the above-mentioned variables, the *subjective norm* was operationalized as the perception of whether important people in the woman’s life (including her mother, husband, and gynecologist) would approve her fasting.

### Validity and reliability

As part of the validity assessment, initial testing of the instrument was done by soliciting feedback from two subject experts followed by a pilot study involving cognitive interviewing from January to February 2020 on a convenient sample of eight pregnant women representing different education and socioeconomic levels and residing in the Beirut area. First, neutral open-ended questions about fasting while pregnant were addressed. Second, the instrument was provided to each woman who was asked to read the questions out loud with the author recording every potential misunderstanding of items. As a result of this testing, the instrument was changed to add the option “not applicable” to several items and rewording the word “Imam” to “Sheikh”. Otherwise, the items were well understood, and the instrument was set as a final draft. Test re-test reliability was carried out using a convenience sample of 20 pregnant women separate from the surveyed sample. Two weeks later, the same instrument was administered to the same group of women. The instrument items included in the analysis showed satisfactory correlation.

### Study population and sample

The target population consisted of Muslim pregnant women in Lebanon. Inclusion criteria required that a participant is Muslim, pregnant, and Lebanese or resides in Lebanon. Individuals showing cognitive or physical impairments that might prevent them from completing the interview were excluded. Potential participants were excluded if they were estimated to give birth before the commencement of Ramadan.

To determine the sample size, it was assumed that only 10% of women who score at the mean value of each TPB construct aggregate would fast. We tested whether women who score one standard deviation above the mean will be 10% more likely to fast. A directional test with an alpha of 0.05 and power = 0.8 with three predictor variables in the model was specified. Results indicated that 160 Muslim pregnant women were needed.

### Data collection

**Fig. 1:**
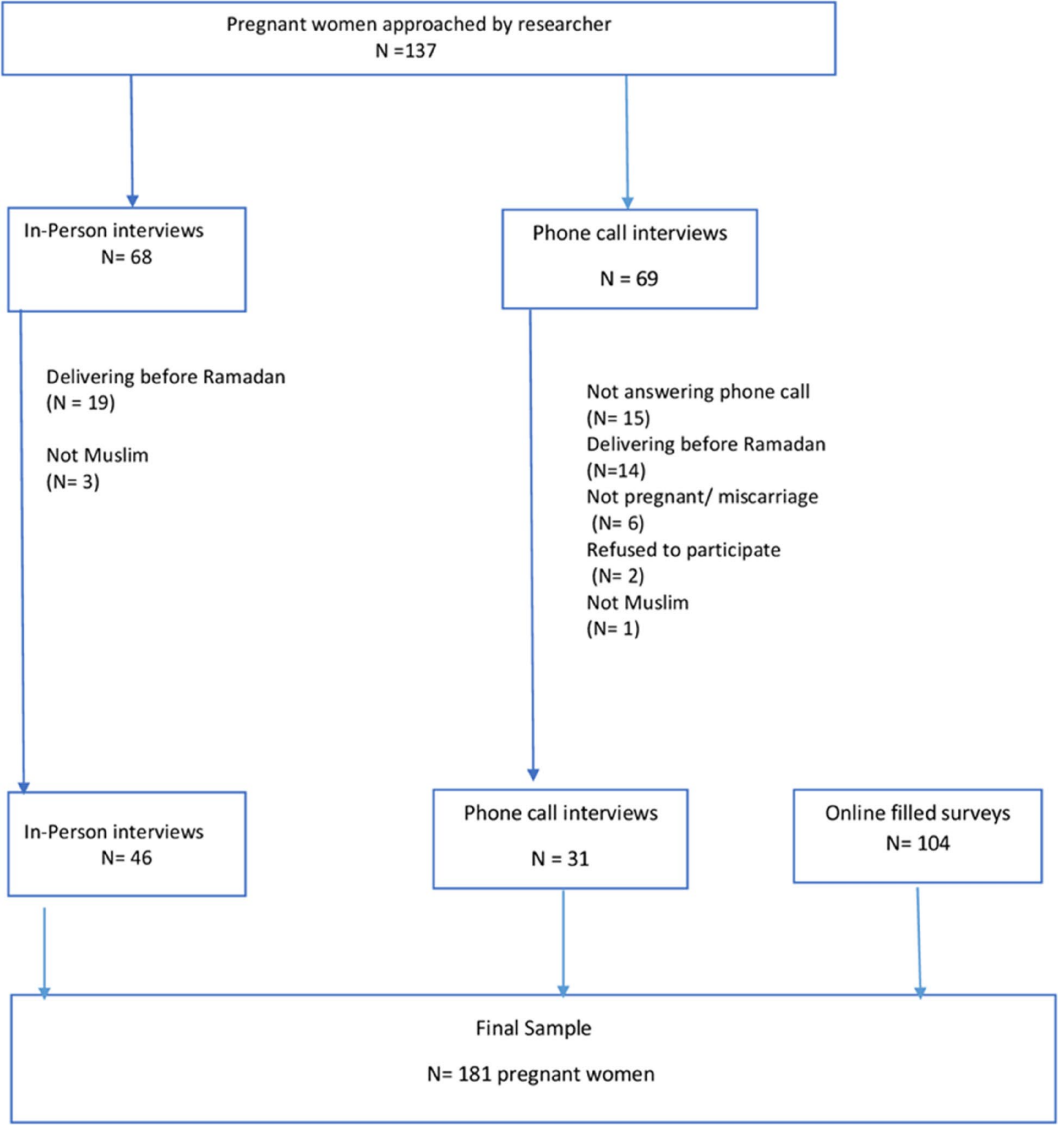
Sample identification and response

The study was carried out on a convenience sample of Muslim pregnant women between February and April 2020. The first phase of data collection started on February 25th, 2020 in six private clinics serving pregnant women in two districts in Beirut. Recruitment started after informing clinic managers of details about the study. The researcher visited the clinics for the data collection on weekdays and Saturdays from 8 A.M to 2 P.M. All Muslim pregnant women visiting the clinics whose expected date of delivery was after the start of Ramadan were invited to participate in this study while waiting their turn for examination by the obstetrician to obtain oral informed consent. The instrument was administered by the interviewer to reduce the effect of literacy levels on interpreting the items. Forty-six surveys were filled at the six clinics. Due to the circumstances surrounding the Severe acute respiratory syndrome coronavirus 2 pandemic, data collection was halted after filling out 46 surveys. Alternative strategies were adopted to reach the required sample size before April 24th, the date when Ramadan began in 2020. An online survey was created using Google Forms with the same series of questions used in the first phase of data collection. The online Google form was shared on two Facebook groups dedicated to issues related to women in all geographic regions of Lebanon. This resulted in an additional 104 surveys, which were filled out online. To achieve a sufficient sample of participants with lower educational levels, we intentionally oversampled an additional 31 pregnant women listed on a database of a non-government organization facility that predominantly serves low-income and underprivileged women in Lebanon. Those women were approached by phone. If the researcher could not reach the woman after the first call attempt, the call was repeated two additional times. Thirty-one surveys were administered by phone in this third and final phase (see Fig. 1).

### Statistical analysis

Descriptive analyses generated frequencies, as well as means, ranges, or standard deviations as relevant were used to describe the characteristics of the sample as well as the dependent and independent variables. An exploratory Bayes tree determined an initial list of variables that separated the intention to fast the best. Those variables were considered in the regression analysis.

Ordinal regression was completed in JMP 14.3® with the intention to fast as the outcome variable (intention to fast all month as reference category) and perceptions of spiritual happiness brought by Ramadan fasting, perceived physical ability, beliefs on Islam guidance, husband’s opinion of importance, mother’s opinion beliefs, Sheikh’s beliefs and their importance, perceived impact on the fetus, and perceived impact on woman’s health as independent variables. Next, a latent class analysis was used to identify different subgroups within participating women that share certain characteristics that could be determined [23] based on the regression results. Class analysis was performed on data from 165 women out of 181 because of missing data.

## Results

### Sample description and intention to fast

The total number of participants resulting from all three phases was 181. The participation rate from in-person interviews among Muslim women meeting the criteria was 100%, while the participation rate resulting from telephone interviews was 94% (31 out of 33). Other details on sample identification and response are available in Fig. 1. The mean sample age was 28 years (SD = 4.5) with 75 % of the participants being 20 to 30 years old. Less than half of the participants had normal body weight recommended to maintain a healthy pregnancy. Nearly two out of three participants held a university degree (68%), but the majority were unemployed (60%). Almost all the participants (99%) did not suffer from diabetes before pregnancy and only 10% were using medications for chronic conditions. The current pregnancy was the first one for 30% of the participants.

More than half of the participants (53%) would have been in the third trimester at the beginning of Ramadan, 35% in the second trimester, and about 12% in the first trimester (see Table 1). Further, 58% of participants intended to fast all days of Ramadan, 22% intended to fast some days and 20% did not intend to fast for any duration. The proportion of women reporting an intention to fast was not closely relevant to the gestational age, but there was a small trend observed. For participants who would have been in the first trimester on Ramadan 1st, 86% reported an intention to fast one or more days, while 80% reported an intention to fast one or more days if they were in their second trimester on Ramadan 1st. The lowest proportion of reported intentions to fast was 78%, which was reported for those who would have been in the third trimester on Ramadan 1st.

**Table 1:** Characteristics and descriptive statistics of participating Muslim pregnant women in Lebanon from February to April 2020 (*N* = 181)

Characteristics	Number (%)^a^	Mean ± SD	Median
Age			
16–19	6 (3)	28 ± 4	27
20–25	55 (31)		
26–30	80 (44)		
31–35	29 (16)		
36–40	11 (6)		
Nationality			
Lebanese	130 (72)		
Syrian or Palestinian	51 (28)		
Educational status			
No education	1 (1)		
Primary education	34 (19)		
Secondary education	23 (12)		
University education	123 (68)		
Employment status			
Not employed	108 (60)		
Employed	73 ( 40)		
Diabetes before pregnancy			
Yes	2 (1)		
No	179 (99)		
Use of medications for chronic conditions			
Yes	18 (10)		
No	163(90)		
Previous pregnancies			
0	54 (30)	1 ± 1	1
1	67 (37)		
2	39 (21)		
3	13 (7)		
4+	8 (5)		
Previous miscarriages			
0	143 (80)	0.2 ± 0.5	0
1	32 (17)		
2+	6 (3)		
Number of children			
0	65 (36)	1 ± 1	1
1	68 (37)		
2	39 (22)		
3+	9 (5)		
Gestational age on Ramadan 1st			
First trimester	21 (12)		
Second trimester	64 (35)		
Third trimester	96 (53)		

^a^Approximation for computed percentages has been performed

### The model predicting fasting intention

The ordinal regression model with all eight predictor variables results indicated that the Perceived impact on the fetus, Sheikh’s beliefs, their importance, and the extent of spiritual happiness were not strong contributors to the model. The Akaike information criterion (AIC) for this model was 207.28 and the Bayesian information criterion (BIC) was 286.86 (see Table 2).

**Table 2:**
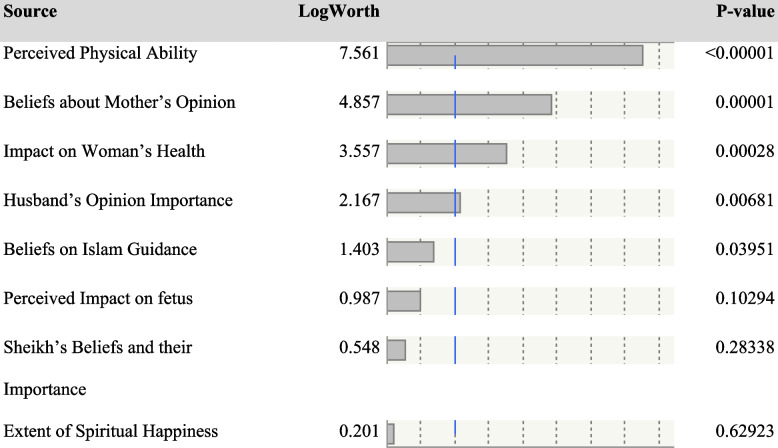
Independent variables and their importance in predicting intent to fast (*N* = 181)

A reduced model was run with perceived physical ability, beliefs on Islam guidance, husband’s opinion importance, mother’s opinion beliefs, and perceived impact on woman’s health. For that model, the AIC was 201.70 and the BIC was 262. 89, both indicating a better fitting model compared to the full model (see Table 3). The generalized *R*-squared for the reduced model was 0.74 indicating a good-fitting model.

**Table 3:** Reduced model showing independent variables and importance in predicting intent to fast and chi-square values (*N* = 181)

Variable	Log worth	*p* value	Df	Chi-square	Prob>Chi-Sq.
Perceived physical ability	14.296	0.00000	4	73.0888502	< 0.0001
Believe mother’s opinion	2.253	0.00558	4	14.6108851	0.0056
Perceived impact on women’s health	2.120	0.00759	4	13.9096018	0.0076
Husband’s opinion importance	1.306	0.04949	4	9.5127471	0.0495
Beliefs in Islam guidance	1.161	0.06898	4	8.70247432	0.0690

### Classes of pregnant women with their intentions to fast

Using intention to fast and the five predictor variables from the final model, a latent class analysis examined 2 through 5 classes. The two clusters had the smallest BIC and four had the smallest AIC. The four-class model was retained as the most parsimonious model. In the four-class model, classes 1 and 2 represented the group of women who had the intention to fast all the month, class 3 represented the group of women who had not the intention to fast whereas class 4 represented the group of women who had the intention to fast for some days only. Class 1 had 38% of the participants, class 2 had 26%, class 3 had 20% and class 4 had 17% of the participants. For women who are not intending to fast, class 3, options do not have an impact, as they are not intending to fast. The only high percentages are believe mother’s opinion, where 76% said their mothers discourage fasting*. Perceived physical ability* also affected the decision within class 3, where 83% stated they were not able to fast. This class is termed “opinion and physical ability affecting the intention not to fast”. For class 4, most were intending to fast for some days and the strongest indicator was their evaluation that they felt they were slightly able to fast. This class is termed “physical ability and the intention to fast for some days”. For class 1, 78% of respondents intended to fast, almost 74% considered themselves able to fast, 69% believe Islam encourages fasting, 45% believe their husband’s opinion is important, 48% believe their mothers encourage fasting, and 64 % do not believe it is harmful at all to their health. This class is termed “opinions, guidance, and health are important for my intention to fast”. For class 2 *beliefs on Islam guidance* are very important. Forty percent of class 2 members chose the highest answer category (extremely important) for *beliefs on Islam guidance*. Together, categories 3 (important) and 4 (extremely important) represent about 93% of Class 2 members. So, in their decisions to fast or not to fast, *beliefs on Islam guidance* represent an important consideration. Moreover, the husband’s and mother’s opinions are important, in the same way. This class is termed “opinions and guidance are very important for my intention to fast”. Class 1 is different, even though women intend to fast, in the decision process, *beliefs on Islam guidance* are not as strong of a factor as they are with class 2 (see Table 4).

**Table 4:** Class analysis by independent variables proportions of response categories ^a^ (*N* = 181)

		Class 1	Class 2	Class 3	Class 4
	Category^b^	0.375	0.258	0.200	0.166
Intention	0	0.0342	0.0022	0.8728	0.0031
	1	0.1865	0.0534	0.1248	0.6975
	2	0.7793	0.9444	0.0024	0.2994
Perceived physical ability	0	0.0014	0.0012	0.8396	0.0047
	1	0.0009	0.0242	0.0614	0.0024
	2	0.1136	0.0253	0.0358	0.8621
	3	0.7398	0.2697	0.003	0.1283
	4	0.1442	0.6796	0.0602	0.0026
Beliefs on Islam guidance	0	0.0776	0.0021	0.2434	0.1516
	1	0.0184	0.0012	0.0617	0.0344
	2	0.17	0.0661	0.2708	0.3536
	3	0.6919	0.5298	0.3925	0.3041
	4	0.0421	0.4009	0.0316	0.1563
Husband’s opinion importance	0	0.1498	0.1107	0.122	0.0028
	1	0.1443	0.002	0.0621	0.0029
	2	0.1636	0.1294	0.1524	0.2317
	3	0.4503	0.3065	0.361	0.3635
	4	0.092	0.4514	0.3025	0.399
Beliefs on mother’s opinion	0	0.1482	0.0717	0.7565	0.3521
	1	0.0984	0.0014	0.0317	0.1434
	2	0.2651	0.055	0.1784	0.2322
	3	0.4865	0.4319	0.0317	0.0496
	4	0.0018	0.44	0.0018	0.2227
Perceived impact on woman’s health	0	0.0009	0.0012	0.1222	0.1456
	1	0.0696	0.0294	0.304	0.2349
	2	0.0791	0.1006	0.2397	0.4333
	3	0.2089	0.0025	0.0616	0.0023
	4	0.6416	0.8662	0.2725	0.1839

^a^Classes 1 and 2 represented the group of women who had the intention to fast all the month, class 3 represented the group of women who had not the intention to fast whereas class 4 represented the group of women who had the intention to fast for some days only

^b^Response categories were as follows: for intention, 0: I have no intention to fast, *1*: I intend to fast for some days, *2* I have the intention to fast all month; For perceived physical ability, *0*: not able to fast, *1*: somewhat able to fast, *2*: slightly able to fast, *3*: able to fast, *4*: extremely able to fast; for belief on Islam guidance, 0: discourages fasting, 1:somewhat encourages fasting, *2*: slightly encourages, fasting, 3: encourages fasting, *4*: extremely encourages fasting; For husband’s opinion importance, 0 not important at all, *1*: somewhat important, *2*: slightly important, *3*: important, *4*: extremely important; for belief on mother’s opinion, 0: discourages fasting, 1:somewhat encourages fasting, *2*: slightly encourages, fasting, *3*: encourages fasting, *4*: extremely encourages fasting; for perceived impact on woman’s health, *0*: not harmful at all, *1*: somewhat harmful, *2*: slightly harmful, *3*: hamrful, *4*: extremely harmful

## Discussion

This study assessed the intention of Muslim pregnant women to fast during Ramadan. The use of the theory of planned behavior along with analytical tools including latent class analysis allowed for a better understanding of the complexity of the factors driving this intention, which appears to be guided by several factors including perceptions of physical ability, opinions of mothers, and husbands with perceptions of the impact on a woman’s health and Islam’s guidance.

One key finding that this study draws attention to is the fact that women who intend to fast all days of Ramadan are not a homogenous group. This group is divided into two distinct subgroups that differ in key variables such as the belief in the extent to which Islam encourages fasting. This appeared to be a strong and key variable for class 2 but a strong factor for class 1. This has implications for communication efforts targeting women in this group. Efforts should be made by clinicians and public health professionals to provide tailored communication messages for pregnant women intending to fast for the whole month of Ramadan that address the drivers behind their decisions starting with how women perceive Islam’s encouragement of their practice.

In this study, almost 58% of the participants had the intention to fast all days of Ramadan. This percentage is higher than what has been observed in other studies conducted in several Asian nations (Pakistan, Singapore, and Indonesia) where the rates of fasting ranged from 14 to 43% [6, 9, 24]. This difference may be partially explained by the conceptual difference between the intention to fast, measured in this study, and the actual fasting behavior, measured in other studies. The analytical model used in this study indicated that women’s belief in their physical ability to fast was very important in predicting the intention of the pregnant to fast during Ramadan. Findings concerning physical ability and fasting are in agreement with a study that was done in Iraq, another Arab country, where almost half of the participants, mentioned that being unable to fast was the most important reason behind not fasting [7]. Along the same lines, the perceived difficulty of fasting during pregnancy after feeling sick and weak was a major reason to skip or stop fasting among pregnant women [9, 25].

The belief of pregnant women that Islam encourages fasting during pregnancy was another influential predictor of the intention to fast. This belief has been explored by other studies where the proportion of women believing that fasting is encouraged by Islam ranged from 38 to 96% [3, 25, 26].

The opinion of the mothers of pregnant women was very important in the decision-making, where the majority of participants who intended to fast believed that their mothers would encourage fasting, and the majority of those not planning to fast mentioned that their mothers would discourage fasting. This is in line with other studies, not related to fasting, that addressed the impact mothers have on their pregnant daughters’ health-related decisions. For example, in a study done in Ghana that assessed the behavior of pregnant teenagers for health-seeking information, mothers were the most important source of information for their pregnant daughters [27]. This might be particularly relevant to the Lebanese cultural context, which emphasizes strong intergenerational family relationships. It may be that the mother is the closest natural and trustworthy “expert” the pregnant woman can access. Perhaps slightly less important, but still an important factor was the husband’s opinion concerning fasting. In this study, 45% of the women intending to fast perceived the opinions of their husbands to be important in guiding their decisions. A study done in Pakistan observed that family members’ opinions on encouraging fasting were particularly trusted by the pregnant participants [3]. Moreover, in Singapore, Joosoph et al. found that the majority of family members and husbands supported the pregnant women and encouraged them to fast [24].

Interestingly, Muslim sheiks were not observed to be contributing to the intention of pregnant women to fast. This is in contrast with a study done in the US state of Michigan, a multicultural setting, where Muslim sheiks influenced pregnant women’s decisions to fast more than family members [5]. The difference might be explained by a sociocultural effect where the difference in cultural context plays a role. Muslims in Muslim minority countries, such as the USA, usually have a sense of community revolving around scheduled gatherings in Mosques, where they can discuss Islamic teachings with religious figures. This would lead to women placing a particular value on sheiks in that setting. Lebanon, on the other hand, is a country with a significant Muslim population where the role of religious figures in this particular issue does not seem to be as prominent.

Health care professionals and, predominantly, the obstetrician or the midwife represent reliable and frequently encountered sources of information to which pregnant women address their health needs and concerns [28]. In this study, however, it was interesting that the obstetrician was not contributing to the intention of pregnant women to fast either directly or indirectly. Communication gaps between health professionals and pregnant women concerning fasting were observed in prior studies, which indicated varying rates of counseling about the issue [5, 25, 29]. In a study done in Pakistan, the majority of participants did not even consult any health professional to assess the safety of fasting during Ramadan [3]. On the other hand, the majority of pregnant women in a study done in Iran consulted their midwife or obstetrician about fasting during Ramadan [4]. Somewhere in between, approximately half of the fasting women in a study done in Germany consulted their obstetricians to discuss fasting status. Several reasons may contribute to the communication gaps described above [25]. Those variations might be related to the fear of pregnant women from discussing this religious issue with their caregivers, and their perceptions that they should be making this decision independently of their providers, as they fear the judgment of their behavior from their providers [3, 4].

Moreover, variation in the rates of consulting obstetricians indicates that primary care providers, pharmacists, and nurses should be proactive in providing pregnant Muslim women with sound advice on fasting as well as encourage them to seek professional help from their obstetricians when fasting during Ramadan is being considered.

### Limitations of the study

As with all studies, this research had both strengths and weaknesses. The study was conducted in Lebanon, a country with a diverse cultural background. The instrument was provided to participants in their native language improving the comprehension of questionnaire items and ultimately improving the quality of data. Theory informed a priori hypotheses that drove the analyses.

Limitations of this project merit discussion. The diversity in the convenience sampling procedure, including online recruitment of pregnant women, resulted in a higher average educational level of participants limiting the generalizability of the study. The study was done in Lebanon, a country with unique diversity. While this represents an addition to the literature, caution should be taken before generalizing to other settings in which Muslims represent an overwhelming majority or minority. The effect of some confounders on the intention to fast cannot be ruled out including the effect of fasting in a previous pregnancy, or if some pregnant women are also breastfeeding. Self-report measures are more likely to result in the overestimation of intention to fast during Ramadan, a socially desirable behavior, that may be seen as an indication of religious piety by some participants. Still, in an attempt to reduce this bias, the survey was self-administered in the online data collection phase, and no identifying information was collected from participants. Mode of administration bias should be considered when participants in the phases involving face-to-face and telephone interviews may be more likely to report fasting. Finally, this study focused on intentions to fast rather than actual fasting, which might change over time with women who initially chose to fast reverse their decision or vice versa.

## Conclusion

Items derived from the TPB constructs helped in developing a model predicting women’s intention to fast during Ramadan, which varied among this study’s participants. Women intending to fast all days of Ramadan are not a homogenous group with perceptions of physical ability differing among that same group. Building on this study, qualitative research with women, their families, and different health professionals would be beneficial for a more in-depth understanding of the relationship between the different variables and intentions to fast. Larger studies should address the applicability of findings reported here in different settings and different countries in addition to including religious fasting of pregnant women adhering to other religions. Educational messages and interventions related to fasting while pregnant may be delivered by individuals with legitimacy among pregnant women such as powerful motherly figures in pregnant women’s communities.

## Data Availability

Relevant data generated or analyzed during this study are included in this published article and its supplementary information files. The data that support the findings of this study are openly available in Mendeley Data at https://data.mendeley.com/datasets/pzfs3zhdp8/1

## Abbreviations

AIC: Akaike information criterion
BIC: Bayesian information criterion
TPB: Theory of planned behavior
